# A Comparison Study of Magnetic Resonance Imaging and Ultrasonography in the Evaluation of Ovarian Lesions With an Emphasis on Ovarian Adnexal Reporting and Data System

**DOI:** 10.7759/cureus.83858

**Published:** 2025-05-10

**Authors:** Nalluri Siddhartha, Vishal Nimbal, Pundalik U Lamani, Tejas Halkude, Pavan Kolekar

**Affiliations:** 1 Radiodiagnosis, Shri BM Patil Medical College Hospital and Research Centre, Vijayapura, IND; 2 Radiodiagnosis, Bijapur Lingayat District Educational (BLDE) Shri BM Patil Medical College Hospital and Research Centre, Vijayapura, IND

**Keywords:** diagnostic performance, histopathology, magnetic resonance imaging, o-rads, ovarian-adnexal reporting and data system, ovarian lesions, ultrasonography

## Abstract

Introduction

Accurate characterization of ovarian lesions is essential for guiding clinical management, influencing decisions on conservative follow-up, medical treatment, or surgical intervention. The Ovarian-Adnexal Reporting and Data System (O-RADS) has emerged as a standardized tool for risk stratification, aiming to improve diagnostic accuracy and management of ovarian adnexal pathologies. This study aimed to compare the diagnostic performance of ultrasonography (USG) and magnetic resonance imaging (MRI) in evaluating ovarian lesions using the O-RADS classification system.

Materials and methods

This prospective observational study was conducted at the Department of Radiodiagnosis, Shri BM Patil Medical College Hospital & Research Centre, Vijayapura, from April 2023 to March 2025. Forty-four patients with clinically suspected ovarian lesions who underwent both USG and MRI, followed by histopathological confirmation, were included. Lesions were classified according to the O-RADS system on both imaging modalities, and findings were correlated with histopathological results. Sensitivity, specificity, positive predictive value, and negative predictive value were calculated for both imaging techniques.

Results

The mean age of patients was 35.7 years, with the majority (29, 65.9%) in the 21-40 years age group. Abdominal pain was the most common clinical presentation (40, 90.9%). Both USG and MRI showed identical lesion morphologies, with multilocular cystic lesions without solid components being the most frequent (13, 29.5%). The distribution of O-RADS scores was similar between the modalities, with most lesions categorized as O-RADS 3 (27(61.4%) on USG and 28 (63.6%) on MRI). Histopathology revealed 35 (79.5%) benign and 9 (20.5%) malignant lesions. A significant correlation was found between O-RADS scores and malignancy (p<0.001), with all O-RADS 3 lesions being benign and all O-RADS 5 lesions being malignant. Both USG and MRI demonstrated identical diagnostic performance, with 100% sensitivity, 95% specificity, 98% positive predictive value, and 97% negative predictive value.

Conclusion

Both ultrasonography and magnetic resonance imaging, when used with the O-RADS classification system, provide excellent and comparable diagnostic accuracy in evaluating ovarian lesions. The significant correlation between O-RADS scores and histopathological findings highlights the system's clinical utility in risk stratification and management decisions. A tiered imaging approach, with ultrasonography as the first-line modality and MRI reserved for specific indications, offers an effective and cost-efficient strategy for managing ovarian pathologies.

## Introduction

Ovarian lesions pose a considerable diagnostic challenge in gynecological imaging, requiring accurate characterization to guide clinical management and improve patient outcomes [1]. With the rising incidence of ovarian pathologies, including benign cysts, borderline tumors, and malignant neoplasms, there is a pressing need for precise imaging techniques that can facilitate early diagnosis and appropriate therapeutic planning [2]. The role of imaging is paramount not only in detecting these lesions but also in differentiating between benign and malignant features, which has significant implications for patient prognosis [3].

Over the past few decades, ultrasonography (USG) and magnetic resonance imaging (MRI) have become cornerstone modalities in the evaluation of adnexal masses [4]. Each has unique strengths that contribute to its respective diagnostic value. Ultrasonography, particularly transvaginal sonography (TVS), is widely utilized as the first-line imaging modality due to its widespread availability, cost-effectiveness, non-invasiveness, and lack of ionizing radiation [5]. It allows for real-time imaging and, when supplemented with color Doppler, provides insight into vascular characteristics that may help distinguish benign from suspicious lesions. However, ultrasonography is inherently operator-dependent and may be limited in its ability to assess complex or deep pelvic lesions, particularly in patients with high body mass index or post-surgical anatomical alterations [6,7].

MRI has emerged as a powerful complementary tool, offering high-resolution, multiplanar imaging and superior soft-tissue contrast. The ability of MRI to assess tissue characteristics using a variety of pulse sequences, including T1-weighted, T2-weighted, and diffusion-weighted imaging, enhances its utility in complex cases [8,9]. MRI is particularly valuable in the characterization of indeterminate adnexal masses initially detected on ultrasound, where it can provide detailed anatomical and functional information that supports more accurate diagnosis and risk stratification [9].

The advent of standardized reporting frameworks has further strengthened the diagnostic process in gynecological imaging. The Ovarian-Adnexal Reporting and Data System (O-RADS), developed by the American College of Radiology, offers a structured approach to the classification of adnexal masses using both ultrasonographic and MRI criteria [10]. O-RADS introduces a standardized lexicon and scoring system that facilitates consistent reporting, improves communication between radiologists and referring clinicians, and ultimately enhances patient care through evidence-based risk assessment [11]. By assigning a risk category from O-RADS 1 (normal) to O-RADS 5 (high risk of malignancy), this system aids in clinical decision-making and helps determine the need for follow-up, intervention, or surgical referral.

Emerging literature supports the utility of both modalities, with ultrasound often proving sufficient for typical benign lesions such as simple cysts, haemorrhagic cysts, and classic endometriomas [12]. Conversely, MRI has demonstrated superior accuracy in evaluating complex or borderline lesions, detecting peritoneal implants, and staging malignancies, particularly in scenarios where ultrasound findings are inconclusive or suggest malignancy [13].

This study aims to perform a comprehensive comparative analysis of MRI and ultrasonography in the evaluation of ovarian lesions, with particular focus on the application of the O-RADS classification system.

## Materials and methods

This hospital-based cross-sectional study was conducted at the Department of Radiology, Bijapur Lingayat District Educational (BLDE, Deemed to be University), Shri BM Patil Medical College Hospital and Research Centre, Vijayapura, between April 2023 and March 2025. The study protocol was approved by the Institutional Ethics Committee (BLDE/IEC/944/2023-24), and written informed consent was obtained from all participants prior to enrolment.

Female patients aged between 12 and 85 years who presented with suspected ovarian masses or had incidental ultrasonographic findings suggestive of ovarian lesions were included in the study. The exclusion criteria for this study were as follows: patients with ultrasound O-RADS scores of 1 and 2 (normal or benign); cases of ectopic pregnancy that were clinically diagnosed and confirmed by ultrasound; patients with cochlear implants, pacemakers, artificial heart valves, or other metallic implants that contraindicate MRI; and patients with a history of claustrophobia that would prevent them from undergoing MRI scanning. Patients who were clinically suspected of having gynecological masses and those referred to the radiology department for evaluation were screened for eligibility. Detailed medical history was obtained from all participants, including presenting complaints, menstrual history, obstetric history, and family history of gynecological malignancies. Using the formula n = (Z² × p × (1 - p)) / d², where Z = 1.96, p = 0.0294, and d = 0.05, the estimated sample size calculated for this study was 44.

All participants underwent transabdominal and/or transvaginal ultrasonography using GE Volusion S8 BT 18 and GE Versana Premier systems (GE HealthCare, Chicago, Illinois). Both gray-scale and color Doppler imaging were utilized to evaluate the ovarian lesions. MRI examinations were performed using a GE Signa Explorer 1.5 TESLA system with standard imaging protocols including T1-weighted, T2-weighted, fat-suppressed T1-weighted, and diffusion-weighted sequences in multiple planes. When indicated, contrast-enhanced sequences were obtained using gadolinium-based contrast agents. The lesions were characterized based on their morphological features, enhancement patterns, and risk stratification scores according to both O-RADS ultrasound (US) and O-RADS MRI systems.

The O-RADS scores for both US and MRI were calculated by assessing the morphological features of the ovarian lesions. Each lesion was assigned a score based on its risk of malignancy, ranging from 1 (benign) to 5 (highly suspicious for malignancy). Interpretation of the O-RADS scores is as follows: O-RADS 1 (normal or benign), O-RADS 2 (benign-appearing), O-RADS 3 (indeterminate, low risk), O-RADS 4 (suspicious, moderate risk), and O-RADS 5 (highly suspicious for malignancy).

**Figure 1:**
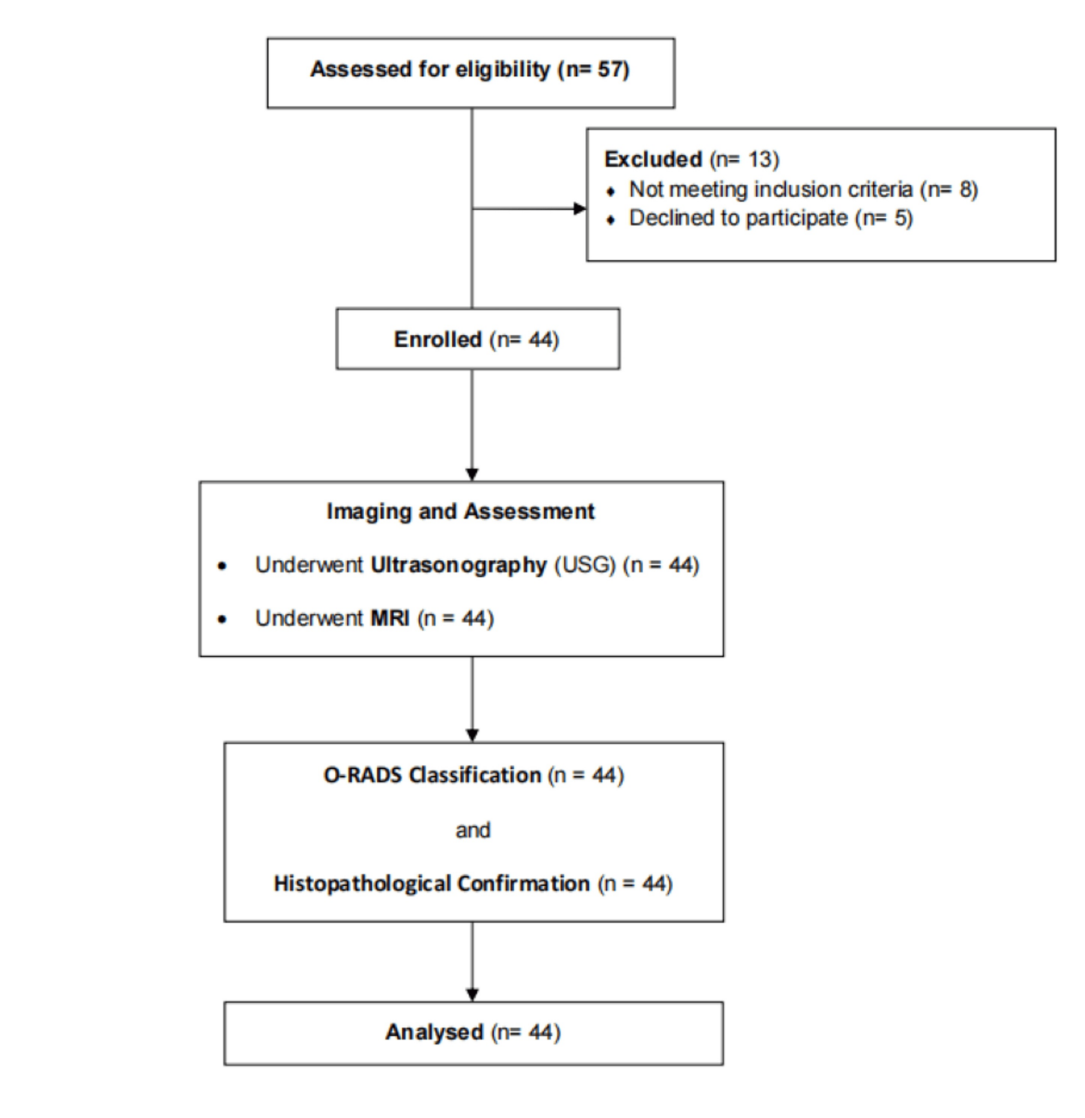
Flow diagram O-RADS - Ovarian-Adnexal Reporting and Data System

For statistical analysis, data were entered into an Excel sheet (Microsoft, Redmond, Washington) and analyzed using SPSS version 26 (IBM Inc., Armonk, New York). The following statistical tests were conducted: descriptive statistics for demographic data and a Chi-squared test to evaluate categorical variables. The sensitivity, specificity, accuracy, positive predictive value (PPV), and negative predictive value (NPV) of MRI and ultrasonography were calculated using standard formulas. Sensitivity was calculated as (true positives) / (true positives + false negatives), specificity as (true negatives) / (true negatives + false positives), accuracy as (true positives + true negatives) / (total population), PPV as (true positives) / (true positives + false positives), and NPV as (true negatives) / (true negatives + false negatives). A p-value of less than 0.05 was considered statistically significant (Figure 1).

## Results

Most patients in the study population were within the reproductive age group, with 29 (65.9%) aged 21-40 years. Other age groups included eight (18.2%) patients aged 41-60 years, five (11.4%) above 60 years, and two (4.5%) between 17-20 years. The predominant clinical symptom was abdominal pain, observed in 40 (90.9%) patients. Less frequent symptoms included backache in two (4.5%) patients, amenorrhea in one (2.3%) patient, and a palpable abdominal mass in one (2.3%) patient. Right-sided ovarian lesions were most common, seen in 23 (52.3%) patients, followed by left-sided lesions in 18 (40.9%) patients and bilateral involvement in three (6.8%) patients (Table 1).

**Table 1:** Distribution of patients according to demographics and clinical presentation Frequency and percentages were calculated for all variables.

Characteristic	Number (%)
Age (years)	
17-20	2 (4.5%)
21-40	29 (65.9%)
41-60	8 (18.2%)
>60	5 (11.4%)
Clinical presentation	
Pain abdomen	40 (90.9%)
Mass per abdomen	1 (2.3%)
Back ache	2 (4.5%)
Amenorrhea	1 (2.3%)
Laterality	
Right	23 (52.3%)
Left	18 (40.9%)
Bilateral	3 (6.8%)

Evaluation of lesion morphology on ultrasonography and MRI revealed a consistent distribution across both imaging modalities. Unilocular cystic lesions without solid components were noted in 10 (22.7%) patients, while unilocular cystic lesions with solid components were found in eight (18.2%) patients. Multilocular cystic lesions without solid components were the most frequent, seen in 13 (29.5%) patients, followed by multilocular cystic lesions with solid components in five (11.4%) patients. Solid lesions were observed in eight (18.2%) patients. O-RADS category 3 was the most commonly assigned risk level, reported in 27 (61.4%) cases on USG and 28 (63.6%) cases on MRI. O-RADS category 4 was noted in nine (20.5%) patients on USG and eight (18.2%) on MRI, while category 5 was assigned in eight (18.2%) patients by both modalities (Table 2).

**Table 2:** Morphological characteristics of ovarian lesions on USG and MRI Frequency and percentages were calculated for all variables. O-RADS - Ovarian-Adnexal Reporting and Data System

Characteristics	USG (%)	MRI (%)
Unilocular cystic without solid component	10 (22.7%)	10 (22.7%)
Unilocular cystic with solid component	8 (18.2%)	8 (18.2%)
Multilocular cystic without solid component	13 (29.5%)	13 (29.5%)
Multilocular cystic with solid component	5 (11.4%)	5 (11.4%)
Solid	8 (18.2%)	8 (18.2%)
O-RADS score		
3	27 (61.4%)	28 (63.6%)
4	9 (20.5%)	8 (18.2%)
5	8 (18.2%)	8 (18.2%)

Histopathological evaluation confirmed benign lesions in 35 (79.5%) patients and malignant lesions in nine (20.5%) patients. Among the benign lesions, serous cystadenoma was the most common, seen in 10 (22.7%) patients, followed by mucinous cystadenoma in six (13.6%), haemorrhagic cyst in five (11.4%), and fibroma in five (11.4%). Other benign diagnoses included endometrioma in four (9.1%), mature teratoma in two (4.5%), hydrosalpinx in two (4.5%), and peritoneal inclusion cyst in one (2.3%) patient. Malignant lesions included serous cystadenocarcinoma in three (6.8%) patients, mucinous cystadenocarcinoma in three (6.8%), endometrioid adenocarcinoma in two (4.5%), and yolk sac tumor in one (2.3%) patient (Table 3).

**Table 3:** Histopathological diagnosis Frequency and percentages were calculated for all variables.

Histopathology	Frequency (%)
Benign lesions (n=35, 79.5%)	
Serous cystadenoma	10 (22.7%)
Mucinous cystadenoma	6 (13.6%)
Haemorrhagic cyst	5 (11.4%)
Fibroma	5 (11.4%)
Endometrioma	4 (9.1%)
Mature teratoma	2 (4.5%)
Hydrosalpinx	2 (4.5%)
Peritoneal inclusion cyst	1 (2.3%)
Malignant lesions (n=9, 20.5%)	
Serous cystadenocarcinoma	3 (6.8%)
Mucinous cystadenocarcinoma	3 (6.8%)
Endometrioid adenocarcinoma	2 (4.5%)
Yolk sac tumor	1 (2.3%)

A statistically significant correlation was observed between O-RADS scoring and final histopathological diagnosis. On USG, 27 (77.1%) benign lesions were assigned O-RADS 3, eight (22.9%) were O-RADS 4, and none were O-RADS 5. In contrast, malignant lesions were predominantly categorized as O-RADS 5 in eight (88.9%) cases, with the remaining one (11.1%) falling under O-RADS 4. Similarly, on MRI, 28 (80%) benign lesions were O-RADS 3, seven (20%) were O-RADS 4, and none were O-RADS 5. Among malignant cases, eight (88.9%) were O-RADS 5, and one (11.1%) was O-RADS 4. The p-value for the association between O-RADS scores and histopathological outcome was <0.001 for both USG and MRI, indicating strong statistical significance (Table 4).

**Table 4:** Correlation of histopathological diagnosis with O-RADS score Frequency and percentages were calculated for all variables. Chi-squared test was applied, and a p-value less than 0.05 was considered significant. O-RADS - Ovarian-Adnexal Reporting and Data System

O-RADS score	Benign (n=35)	Malignant (n=9)	Chi-squared value	p-value
USG				
3	27 (77.1%)	0	38.5368	<0.001
4	8 (22.9%)	1 (11.1%)		
5	0	8 (88.9%)		
MRI				
3	28 (80%)	0	38.6222	<0.001
4	7 (20%)	1 (11.1%)		
5	0	8 (88.9%)		

Both ultrasonography and MRI demonstrated excellent diagnostic performance. Sensitivity for detecting malignancy was 100% for both modalities, with a specificity of 95%. The positive predictive value (PPV) was 98% and the negative predictive value (NPV) was 97% for both USG and MRI, indicating high diagnostic reliability (Table 5).

**Table 5:** Diagnostic performance of USG and MRI

Diagnostic parameter	USG	MRI
Sensitivity	100%	100%
Specificity	95%	95%
Positive predictive value	98%	98%
Negative predictive value	97%	97%

## Discussion

Our study demonstrated excellent concordance between ultrasonography (USG) and magnetic resonance imaging (MRI) in characterizing the morphology of ovarian lesions, with both modalities identifying identical proportions across lesion subtypes. The most frequently observed lesion morphology was multilocular cystic without solid components (29.5%), followed by unilocular cystic lesions without solid components (22.7%). This distribution mirrors findings from Thomassin-Naggara et al., who reported that multilocular cystic lesions accounted for approximately 31% of ovarian masses in their prospective multicentre study [13]. The consistent morphological classification across both imaging techniques reinforces the diagnostic reliability of these modalities, particularly when combined with structured reporting systems.

The distribution of O-RADS scores in our cohort was similarly aligned between USG and MRI, with the majority of lesions classified as O-RADS 3 (61.4% on USG and 63.6% on MRI). This reflects the expected clinical spectrum, where intermediate-risk lesions predominate. These results are in line with findings from Andreotti et al., who evaluated 1194 adnexal masses and reported comparable distributions: O-RADS 3 (58.7%), O-RADS 4 (23.1%), and O-RADS 5 (18.2%) [14]. The similarity in scoring patterns further supports the reproducibility of the O-RADS system across imaging modalities and diverse clinical settings.

A key observation in our study was the strong correlation between O-RADS categories and histopathological outcomes, with statistically significant p-values (<0.001) for both imaging modalities. All lesions categorized as O-RADS 3 were confirmed benign, and all lesions classified as O-RADS 5 were malignant. This finding corroborates previous validation studies. Amor et al. reported malignancy rates of 1.4%, 27%, and 85.5% for O-RADS 3, 4, and 5, respectively, using ultrasound [15]. Similarly, Thomassin-Naggara et al. reported malignancy rates of 2%, 33%, and 92% for O-RADS 3, 4, and 5, respectively, using MRI [16]. Our slightly higher specificity for O-RADS 3 and comparable results for O-RADS 5 affirm the applicability and robustness of the O-RADS classification in our setting.

In comparing the diagnostic performance of USG and MRI, both modalities demonstrated exceptional accuracy, with 100% sensitivity, 95% specificity, 98% positive predictive value, and 97% negative predictive value. Although MRI is traditionally viewed as superior in terms of soft tissue characterization due to its multiparametric imaging capabilities, our results suggest that high-quality ultrasonography, when performed by experienced radiologists and guided by standardized criteria, can achieve equivalent diagnostic accuracy. These findings are consistent with those of Valentini et al., who found nearly identical diagnostic accuracies between USG (92%) and MRI (93.5%) in characterizing complex adnexal masses when interpreted by experts [17]. Therefore, MRI should be employed selectively, primarily in cases where ultrasound findings are indeterminate or where additional anatomical detail may influence clinical decision-making.

The strengths of this study include its prospective design, the use of both USG and MRI in all cases, and the application of the standardized O-RADS system for lesion categorization. Histopathological confirmation in all patients provided a robust gold standard for diagnostic correlation, enhancing the validity of the results. Furthermore, conducting the imaging interpretation in a blinded manner helped reduce potential bias.

However, several limitations must be acknowledged. The relatively small sample size may limit the generalizability of the findings. Additionally, this was a single-center study, and operator expertise may have influenced the diagnostic performance, particularly for ultrasonography. Larger, multicentre studies with broader demographic representation are warranted to further validate these findings and confirm the reproducibility of O-RADS scoring in diverse clinical settings.

## Conclusions

This study reinforces the diagnostic reliability and clinical value of both ultrasonography and magnetic resonance imaging in the evaluation of ovarian lesions when guided by the Ovarian-Adnexal Reporting and Data System (O-RADS). The identical diagnostic performance of the two modalities-demonstrating 100% sensitivity, 95% specificity, and high predictive values-highlights the strength of standardized reporting in achieving consistent, reproducible results. The strong correlation between O-RADS scores and histopathological outcomes underscores the system's effectiveness in risk stratification, particularly the excellent specificity of O-RADS 3 for benign lesions and high malignancy correlation with O-RADS 5. Given the perfect concordance in lesion morphology assessment, ultrasonography, when expertly performed, emerges as a highly capable first-line modality, with MRI best reserved for indeterminate or complex cases. These findings advocate for a rational, tiered imaging approach supported by standardized classification to optimize resource utilization, improve diagnostic confidence, and guide evidence-based clinical management of ovarian pathologies.
